# Presenilin Is the Molecular Target of Acidic γ-Secretase Modulators in Living Cells

**DOI:** 10.1371/journal.pone.0030484

**Published:** 2012-01-06

**Authors:** Thorsten Jumpertz, Andreas Rennhack, Julia Ness, Sandra Baches, Claus U. Pietrzik, Bruno Bulic, Sascha Weggen

**Affiliations:** 1 Department of Neuropathology, Heinrich-Heine-University, Duesseldorf, Germany; 2 Research Group Chemical Biology of Neurodegenerative Diseases, Center of Advanced European Studies and Research, Bonn, Germany; 3 Molecular Neurodegeneration Group, Institute of Pathobiochemistry, University Medical Center of the Johannes Gutenberg-University, Mainz, Germany; University of Regensburg, Germany

## Abstract

The intramembrane-cleaving protease γ-secretase catalyzes the last step in the generation of toxic amyloid-β (Aβ) peptides and is a principal therapeutic target in Alzheimer's disease. Both preclinical and clinical studies have demonstrated that inhibition of γ-secretase is associated with prohibitive side effects due to suppression of Notch processing and signaling. Potentially safer are γ-secretase modulators (GSMs), which are small molecules that selectively lower generation of the highly amyloidogenic Aβ42 peptides but spare Notch processing. GSMs with nanomolar potency and favorable pharmacological properties have been described, but the molecular mechanism of GSMs remains uncertain and both the substrate amyloid precursor protein (APP) and subunits of the γ-secretase complex have been proposed as the molecular target of GSMs. We have generated a potent photo-probe based on an acidic GSM that lowers Aβ42 generation with an IC_50_ of 290 nM in cellular assays. By combining *in vivo* photo-crosslinking with affinity purification, we demonstrated that this probe binds the N-terminal fragment of presenilin (PSEN), the catalytic subunit of the γ-secretase complex, in living cells. Labeling was not observed for APP or any of the other γ-secretase subunits. Binding was readily competed by structurally divergent acidic and non-acidic GSMs suggesting a shared mode of action. These findings indicate that potent acidic GSMs target presenilin to modulate the enzymatic activity of the γ-secretase complex.

## Introduction

Alzheimer's disease (AD) is the most common age-related neurodegenerative disease with an estimated 5.4 million patients in the USA [1]. It is believed that progressive neurodegeneration and cognitive decline in AD are triggered by oligomerization and accumulation of toxic amyloid-β (Aβ) peptides in the brain. The amyloid hypothesis is strongly supported by the analysis of early-onset familial forms of AD (FAD), which has demonstrated that modest overproduction of the oligomerization-prone Aβ42 peptides in the brain is sufficient to cause AD with complete penetrance [2].

The intramembrane-cleaving protease γ-secretase is responsible for the last step in the proteolytic release of Aβ42 peptides from the amyloid precursor protein (APP), and is a principal therapeutic target in AD [3]. γ-Secretase is a multi-subunit aspartyl protease with the presenilin (PSEN) proteins, either PSEN1 or PSEN2, as its catalytic core. PSEN proteins encompass nine transmembrane domains (TMDs) and are endoproteolytically cleaved during assembly of the γ-secretase complex into N- and C-terminal fragments that remain non-covalently associated. The PSEN fragments are incorporated together with three accessory proteins, nicastrin, anterior pharynx defective-1 (APH-1) and presenilin enhancer-2 (PEN-2), into high molecular weight complexes that display proteolytic activity [3]. Two critical aspartate residues in TMD6 and TMD7 of PSEN form the active center of γ-secretase [4]. How γ-secretase accomplishes the hydrolysis of peptide bonds in the hydrophobic environment of the membrane is only partially understood. TMDs 6 and 7 of PSEN around the catalytic aspartate residues form a hydrophilic cavity within the membrane that may allow access for water molecules required to hydrolyze peptide bonds [5], [6]. In addition, it appears that the substrate initially binds to a distinct substrate-binding (docking) site on the outer surface of the γ-secretase complex and is subsequently transported into the active site and cleaved [7].

Despite the limited knowledge of the catalytic mechanism of γ-secretase and the lack of high-resolution structural data, a large number of small molecule γ-secretase inhibitors (GSIs) with excellent potency and *in vivo* properties have been developed [8]. Unfortunately, both preclinical and clinical studies have demonstrated that inhibition of γ-secretase is associated with prohibitive side effects due to suppression of Notch processing and signaling [8], [9]. γ-Secretase modulators (GSMs) are small molecules that selectively lower generation of the highly amyloidogenic Aβ42 peptides but spare Notch processing, and might be a safer alternative to GSIs [10]. The first GSMs were described in the class of non-steroidal anti-inflammatory drugs (NSAIDs) [11]. However, these NSAID-type GSMs suffered from low potency and brain penetration. Recently, GSMs with nanomolar potency and favorable pharmacological properties have been reported in two major structural classes: carboxylic acids with structural similarities to NSAIDs (acidic GSMs) and compounds based on bridged aromatics that do not resemble NSAIDs and lack a carboxylic acid group (non-acidic GSMs) [10].

Several studies have confirmed that GSMs modulate enzyme activity in cell-free γ-secretase assays [12], [13], [14]. However, aside from this compelling evidence that GSMs directly interact with the γ-secretase complex, their molecular mechanism remains largely undefined. Photo-affinity labeling studies have identified PSEN as the molecular target of different classes of GSIs [7], [15], [16], [17]. In contrast, previous attempts to elucidate the molecular target of GSMs have produced conflicting results and both the substrate amyloid precursor protein (APP) and subunits of the γ-secretase complex have been proposed [18], [19], [20], [21]. Using a novel photo-probe based on a potent piperidine GSM, we have now demonstrated that PSEN is the molecular target of acidic GSMs in living cells.

## Results

**Figure 1:**
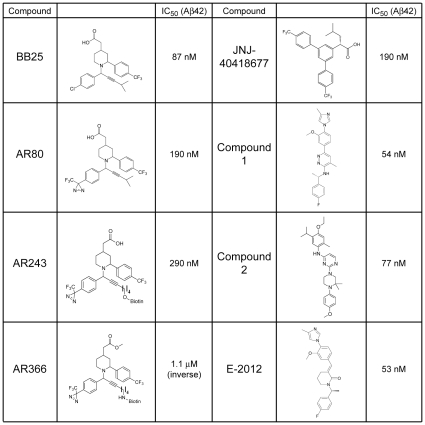
Chemical structures and Aβ42-lowering activities of γ-secretase modulators used in this study. IC_50_ values were determined after treatment of CHO cells with stable co-expression of wild type human APP and PSEN1 as described in the Experimental Procedures. Note that AR366 is an inverse γ-secretase modulator with Aβ42-raising activity.

**Figure 2:**
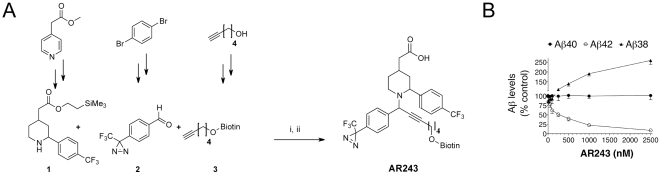
Synthesis and bioactivity of the γ-secretase modulator photo-probe AR243. (**A**) The γ-secretase modulator (GSM) photo-probe AR243 was synthesized by a copper(I)-catalyzed three-component coupling between piperidine-derivative **1**, diazirinyl-benzaldehyde **2**, and biotin-alkyne **3**. The intermediate was then de-protected to yield the free acidic photo-probe. i: CuBr, molecular sieves, Tol. 60°C, 2 d. ii: TBAF, THF, r.t., 2 h. (**B**) CHO cells with stable co-expression of wild type APP and wild type PSEN1 were treated with increasing concentrations of the photo-probe AR243 or DMSO vehicle, and Aβ levels in conditioned media were quantified by sandwich immunoassay. AR243 caused a dose-dependent decrease in Aβ42 levels with a concomitant increase in Aβ38 levels, confirming its bioactivity as a potent GSM with an IC_50_ for Aβ42 reduction of 290 nM.

We recently presented the GSM BB25, derived from Merck patent WO20006043064, which lowered Aβ42 levels in cellular assays with an IC_50_ = 87 nM and served as a starting point for the synthesis of our GSM photo-probe AR243 [22] (Figure 1). In this compound class, potent acidic GSMs such as GSM-1 with good bioavailability, brain penetration and Aβ42-lowering efficacy in AD mouse models have been described [23], [24], [25]. Because of inconsistent results in previous attempts to establish the GSM target [18], [19], we applied strict criteria to the design of the photo-probe. These included potency in the nanomolar range, small size to avoid steric hindrance, and incorporation of a diazirine photo-reactive group to allow cross-linking in living cells. Structure activity relationships that we obtained during the synthesis of BB25 enabled us to synthesize the photo-probe AR80 matching these criteria (Figure 1). The preserved GSM activity of AR80 (IC_50_ = 190 nM) confirmed the tolerance of the scaffold for the introduction of the diazirine group on the para position of the lower aromatic ring, providing a non-invasive probe due to near complete similarity with the parent compound BB25. Subsequently, the biotin moiety for affinity purification was placed on the alkynyl side chain, yielding the photo-probe AR243. Briefly, compounds were synthesized via a copper-catalyzed three component coupling reaction between a racemic piperidine **1**, a diazirinyl-benzaldehyde **2** and a terminal alkyne **3** (Figure 2A).

To establish the bioactivity of the photo-probe, CHO cells with stable overexpression of wild type APP and PSEN1 were treated with AR243 and Aβ levels in culture supernatants were measured by sandwich ELISA. This demonstrated that AR243 behaved like a typical GSM and lowered Aβ42 levels with an IC_50_ = 290 nM, comparable to the potency of the parent compound BB25 (Figure 2B).

**Figure 3:**
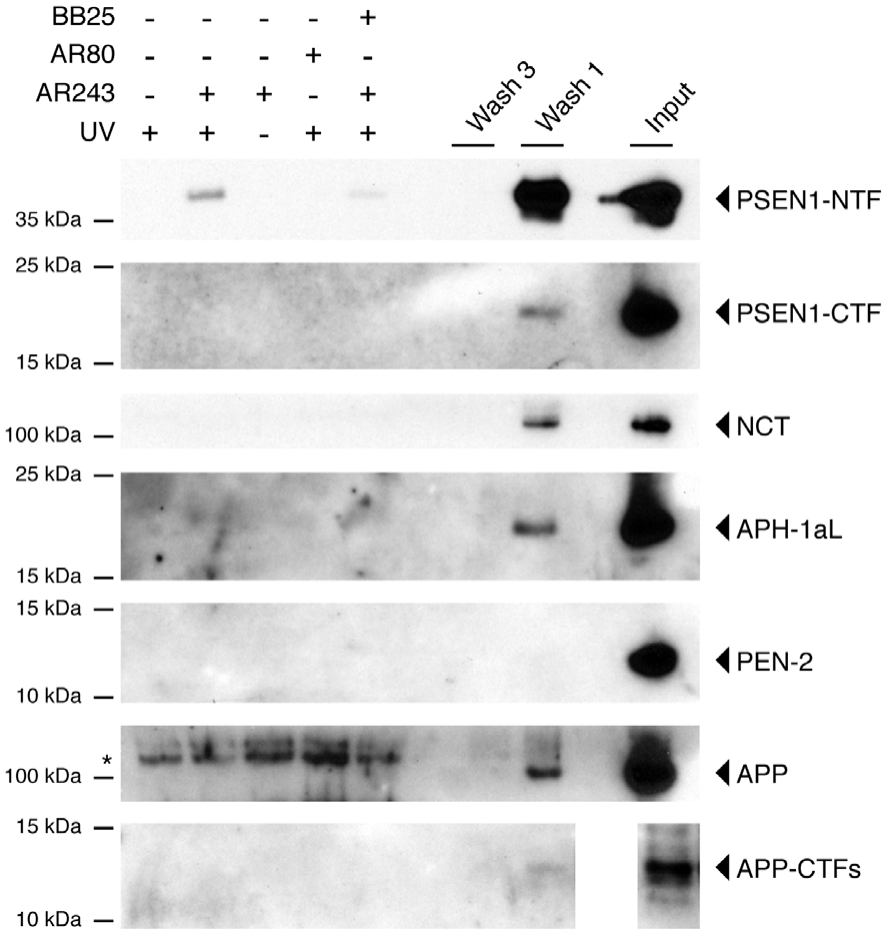
The acidic γ-secretase modulator photo-probe AR243 targets the N-terminal fragment of PSEN1. Cellular membranes were prepared from neuronal N2a-ANPP cells, solubilized in 0.25% CHAPSO, and incubated with 0.5 µM of the photo-probe or DMSO vehicle. After UV irradiation, putative target proteins were affinity purified using streptavidin beads as described in the Experimental Procedures. Western blotting of the purified material demonstrated that the GSM photo-probe AR243 bound the N-terminal fragment of PSEN1 (PSEN1-NTF). Omitting the UV irradiation or incubation of membranes with a 20-fold higher concentration of compound AR80, which lacked the biotin moiety, did not result in a Western blotting signal. Adding an excess of the parent compound BB25 (100 µM) during incubation with the photo-probe efficiently reduced the labeling signal. Western blotting did not demonstrate binding of the photo-probe AR243 to the C-terminal fragment of PSEN1 (PSEN1-CTF) or any of the remaining γ-secretase subunits APH-1, PEN-2 and Nicastrin. Likewise, neither full-length APP nor its membrane-bound C-terminal fragments (APP-CTFs) were labeled by the photo-probe. The asterisk depicts an unspecific protein band of slower mobility that is present under all conditions. Material that was bound non-specifically to the streptavidin beads is visible in the supernatant of the first washing steps but is completely removed after the third wash. Input represents 0.02% (PSEN1-NTF) or 0.2% (all other proteins) of the total membrane material.

**Figure 4:**
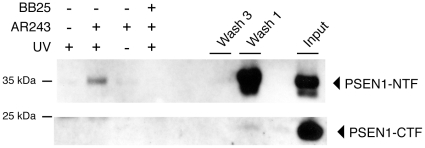
The γ-secretase modulator photo-probe AR243 targets endogenous PSEN1 in human HEK293T cells. Photo-affinity labeling studies were performed with membrane preparations from human HEK293T cells as described in Fig. 1. Western blotting of purified target proteins demonstrated labeling of endogenous PSEN1-NFT but not PSEN1-CTF by AR243. Co-incubation with an excess of parent compound BB25 (100 µM) caused displacement of the photo-probe, demonstrating specificity of the binding to PSEN1-NTF. Input represents 0.02% of the total membrane material.

To identify the molecular target of AR243, cellular membranes were prepared from neuronal N2a-ANPP cells, which stably overexpress APP and all four subunits of the γ-secretase complex [26], and solubilized in 0.25% CHAPSO. These conditions have previously been shown to preserve the integrity and bioactivity of the γ-secretase complex [7], [16]. Subsequently, membrane preparations were incubated with 0.5 µM of the photo-probe or DMSO vehicle, UV irradiated, and putative target proteins were affinity purified using streptavidin beads. The purified material was resolved on SDS gels and probed by Western blotting with antibodies against the four subunits of the γ-secretase complex or the substrate APP. Western blotting with the monoclonal antibody PSN2 demonstrated that the GSM photo-probe AR243 bound the N-terminal fragment of PSEN1 (PSEN1-NTF) (Figure 3). Omitting the UV irradiation or incubation of membranes with a 20-fold higher concentration of compound AR80 (IC_50_ = 190 nM), which was structurally identical to the photo-probe but lacked the biotin moiety, did not result in a Western blotting signal. As expected, adding an excess of the parent compound BB25 (100 µM) during incubation of membrane preparations with the photo-probe efficiently reduced the labeling signal. Western blotting for the C-terminal fragment of PSEN1 (PSEN1-CTF) and the remaining γ-secretase subunits APH-1, PEN-2 and Nicastrin did not demonstrate binding by the photo-probe AR243. Likewise, neither APP nor its membrane-bound C-terminal fragments (APP-CTFs), which are generated after shedding of the APP ectodomain and are the proximate γ-secretase substrates, were labeled by the photo-probe (Figure 3). Next, we sought to confirm binding of the GSM photo-probe to endogenously expressed PSEN1-NTF. Since the PSN2 antibody is specific for human PSEN1, we used membrane preparations from human HEK293T cells and performed the photo-affinity labeling as described above. Western blotting demonstrated labeling of endogenous PSEN1-NFT but not PSEN1-CTF by AR243 (Figure 4). Control experiments including displacement of the photo-probe by an excess of parent compound BB25 demonstrated specificity of the binding to PSEN1-NTF.

**Figure 5:**
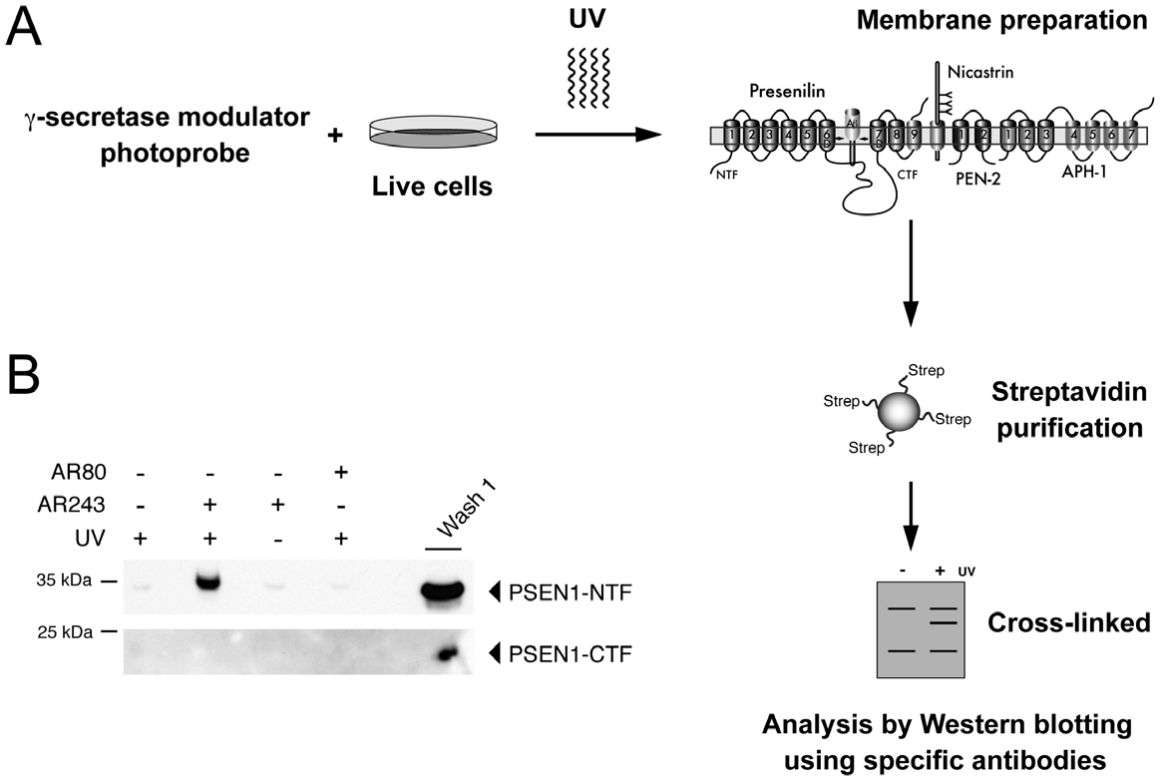
PSEN is the molecular target of the γ-secretase modulator photo-probe AR243 in living cells. (**A**) Schematic illustration of the photo-affinity labeling strategy in living cells. The active GSM photo-probe contains a diazirine photo-reactive group and a biotin moiety for affinity purification. Live cells were treated with the photo-probe and subsequently irradiated in two steps at 365 and 302 nm. Note that the use of long-wave UV light ≥300 nm did not induce acute cellular toxicity. Upon UV irradiation, the photo-probe forms a stable adduct with its specific target. Cellular membranes were prepared and solubilized, and target proteins of the photo-probe were captured and affinity purified using streptavidin-functionalized beads. After extensive washing, the purified material was eluted and resolved on SDS gels and probed by Western blotting. (**B**) N2a-ANPP cells were incubated with 200 nM of AR243 or DMSO vehicle, and target proteins of the GSM photo-probe were purified as described above. Western blotting with the antibody PSN2 demonstrated binding of the photo-probe to PSEN1-NTF. Incubation of cells with 20 µM of compound AR80 that lacked the biotin moiety, or omission of UV irradiation did not result in a labeling signal. Consistent with the *in vitro* labeling results, Western blotting with an antibody against the PSEN1-CTF did not demonstrate binding of the photo-probe confirming that the binding site is located within the PSEN1-NTF.

Diazirine photo-reactive groups are activated by long-wave UV light ≥300 nm, which permits the use of diazirine-based photo-probes in living cells without the induction of acute cellular toxicity. Hence, to provide critical confirmation for our *in vitro* photo-labeling results, we treated N2a-ANPP cells with 0.2 µM of the photo-probe AR243 or DMSO vehicle and irradiated the live cells with UV light (Figure 5A). Subsequently, cellular membranes were prepared and target proteins of the photo-probe were captured and analyzed as described for the cell-free experiments above. Western blotting with PSN2 antibody demonstrated efficient labeling of PSEN1-NTF, which was abolished by omitting UV irradiation (Figure 5B). As expected, incubation of cells with DMSO or a 100-fold higher concentration of compound AR80 lacking the biotin moiety did not result in a positive labeling signal. In accordance with the labeling results *in vitro*, Western blotting did not demonstrate binding of the photo-probe AR243 to PSEN1-CTF *in vivo* (Figure 5B). None of the compound treatments nor the UV irradiation caused cellular toxicity as monitored by alamarBlue**®** reagent (data not shown). However, because we observed dose-dependent cytotoxicity with BB25 concentrations >10 µM, we did not pursue competition experiments in living cells.

**Figure 6:**
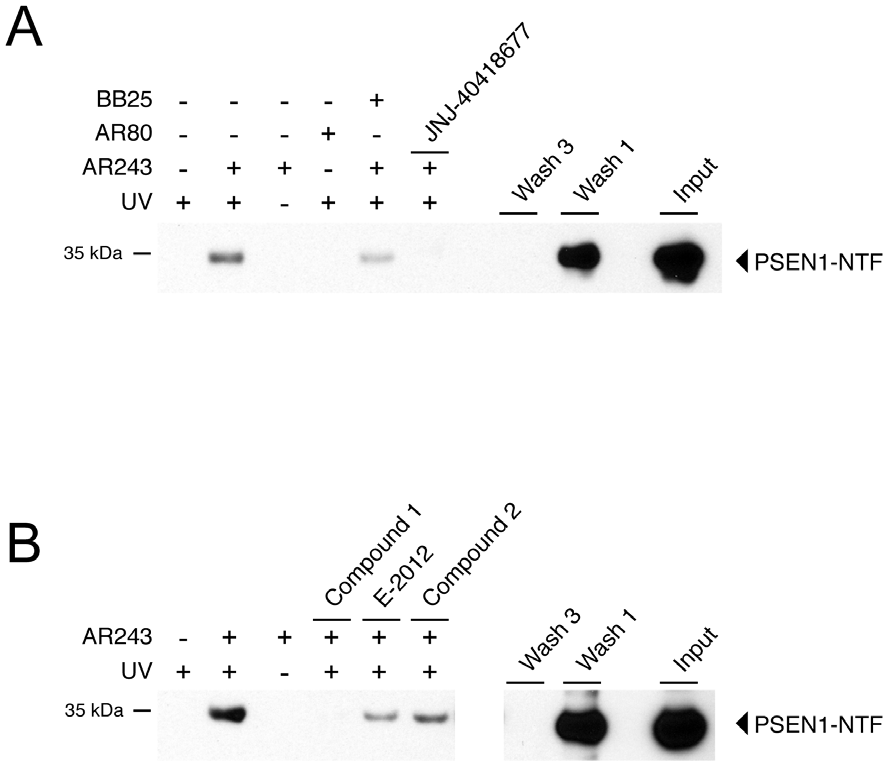
Structurally divergent acidic and non-acidic γ-secretase modulators compete binding of the photo-probe AR243 to the N-terminal fragment of PSEN1. Competition experiments were performed under cell-free conditions with membrane preparations from N2a-ANPP cells. Membranes were co-incubated with 0.5 µM of the photo-probe AR243 and 100 µM of acidic or non-acidic GSM competitor compounds. (A) The acidic GSM JNJ-40418677 abolished binding of the photo-probe to PSEN1-NTF. (B) Three non-acidic GSMs competed binding of the photo-probe to variable degrees. Compound 1 reduced binding of the photo-probe as effectively as the acidic compound JNJ-40418677. Compound 2 and E-2012 only partially competed binding of the photo-probe to PSEN1-NTF. Input represents 0.02% of the total membrane material.

To investigate whether a binding site within the PSEN1-NTF is unique to our photo-probe or likely common to potent second-generation GSMs, we performed competition experiments with other acidic and non-acidic GSMs in our cell-free system using membranes from N2a-ANPP cells. JNJ-40418677, an acidic GSM with considerable structural diversity to the photo-probe and an IC_50_ = 290 nM, abolished binding of the photo-probe to PSEN1-NTF (Figure 6A). In addition, three non-acidic GSMs with IC_50_ values below 100 nM competed binding of the photo-probe to variable degrees. Whereas the non-acidic compound 1 reduced binding of the photo-probe as effectively as the acidic compound JNJ-40418677, compound 2 and E-2012 caused more modest reductions (Figure 6B). Hence, in aggregate, the results of our competition studies indicate that acidic and non-acidic GSMs might share an overlapping binding site within the PSEN1-NTF. Finally, earlier studies had demonstrated that derivatization of acidic GSMs at the carboxylic acid group resulted in inverse GSMs that increase Aβ42 levels and concomitantly decrease the production of shorter peptides such as Aβ38 [27]. Accordingly, through esterification we obtained the inverse GSM photo-probe AR366, which increased Aβ42 levels in our cellular assay with an IC_50_ = 1.1 µM while dose-dependently lowering Aβ38 levels. Incubation of membranes from N2a-ANPP cells with 500 nM of AR366 also resulted in photo-affinity labeling of PSEN1-NTF suggesting that acidic GSMs and inverse GSMs either target the same or a closely related binding site (Figure S1). In conclusion, our findings identify PSEN as the molecular target of potent acidic GSMs using established cell-free conditions and living cells.

## Discussion

Limiting Aβ production and accumulation in the brain through inhibition of γ-secretase remains a rational approach for treatment or prevention of AD. However, GSIs that function as classical protease inhibitors and reduce enzymatic activity have displayed mechanism-based toxicity, which appears to be largely attributable to suppression of Notch processing [8]. In contrast, GSMs selectively lower production of the amyloidogenic Aβ42 peptides and have been shown to spare processing of Notch [11], [13].

While chemical development has yielded a second generation of GSMs with nanomolar potencies and improved brain permeability, the understanding of the molecular mechanism of GSMs is still rudimentary [10]. Photo-affinity labeling and cross-competition studies have demonstrated that, depending on their chemical structure and mechanism of action, GSIs target either the active site of γ-secretase, the docking site or an allosteric site, which are all located within the PSEN proteins [7], [15], [16], [17]. Surprisingly, previous photo-affinity labeling studies have suggested that the binding site of NSAID-type GSMs resides within the substrate APP and not within PSEN or one of the three other γ-secretase subunits [19]. In these studies, photo-probes based on the NSAID-type GSM flurbiprofen and the inverse GSM fenofibrate with benzophenone as a photo-reactive moiety were shown to bind APP, either in the presence of purified recombinant APP-CTFs or in crude membrane preparations of APP overexpressing cells. However, the concept of GSMs targeting the substrate APP contradicted important earlier observations, which suggested interaction of GSMs with the enzyme complex and specifically with PSEN. (i) Kinetic studies have demonstrated non-competitive inhibition for NSAID-type GSMs in γ-secretase *in vitro* assays [12], [13]. This indicated that the inhibitory effect of GSMs on Aβ42 production could not be overcome by increasing substrate concentrations, which would be expected for compounds with a primary binding site within the substrate. (ii) While GSMs do not impair the release of the intracellular signaling domain from Notch-1, which is critical for Notch signaling, it has been convincingly demonstrated that NSAID-type GSMs modulate γ-secretase cleavage events in the middle of the Notch-1 TMD [28]. These are analogous to the cleavage events in the APP TMD that generate the Aβpeptides suggesting that proteolytic processing of at least APP and Notch is affected in a similar manner, thereby questioning the specificity of GSMs for APP. (iii) The aspartyl intramembrane-cleaving protease signal peptide peptidase (SPP) has been demonstrated to harbor a binding site for NSAID-type GSMs [29], [30]. SPP is homologous to PSEN but functions as a homodimer without accessory proteins, and γ-secretase and SPP substrates do not show any overlap. This suggested that γ-secretase contains a binding site for GSMs that is conserved in SPP and, accordingly, should be present in PSEN [29], [30]. (iv) Circumstantial evidence for the interaction of GSMs with the enzyme has been provided by the observation that some FAD-associated PSEN but not APP mutations render cells resistant to GSMs [22], [25], [31], [32].

Using a novel GSM photo-probe with nanomolar potency, we have now identified the PSEN1-NTF as the molecular target of acidic GSMs. In contrast, we did not observe binding of the photo-probe to the substrate APP or any of the other γ-secretase subunits. Importantly, our GSM photo-probe with a diazirine photo-reactive moiety allowed us to confirm targeting of the PSEN1-NTF in living cells. A photo-probe with inverse Aβ42-raising activity similarly labeled PSEN1-NTF indicating that acidic GSMs and inverse GSMs likely target similar or identical binding sites. Under established cell-free conditions that preserve the integrity of the γ-secretase complex, binding of the photo-probe to PSEN1-NTF was readily competed by unrelated acidic and non-acidic GSMs indicating that our findings are not limited to the specific class of piperidine GSMs. These findings are to some extent consistent with a recent study by Kounnas et al. [18]. In this study, a potent non-acidic GSM was coupled to a solid support and used for affinity chromatography. The affinity ligand retained predominantly the γ-secretase subunit PEN-2 and smaller amounts of PSEN1-NTF and PSEN1-CTF from cellular lysates [18]. However, this was only observed when cells were solubilized in 1% Triton X-100 or β-D-maltoside, detergent conditions that do not support γ-secretase activity, whereas solubilization in CHAPSO did not result in PEN-2 retention. Furthermore, it was not investigated whether the affinity ligand preserved its GSM activity after immobilization [18]. Notably, our results are entirely consistent and complementary to a recent study by Ebke and colleagues, who identified the PSEN1-NTF as the molecular target of a distinct class of GSMs, non-acidic aminopyrimidines [21]. During preparation of this manuscript, we further learned that Ohki and colleagues had identified the PSEN1-NTF as the target of acidic, piperidine GSMs using a photo-probe that is similar but structurally clearly distinct from our probe [33].

The reasons for the divergent results of photo-affinity labeling studies with NSAID-type GSMs and the second-generation piperidine GSMs are not known. However, given the low potency of the parent compounds, the photo-probes based on NSAID-type GSMs were applied at high concentrations (10–100 µM) in the photo-affinity labeling studies, raising concerns for non-specific interactions. Moreover, many GSMs share common structural features with known aggregation inhibitors such as extended conjugated aromatics and anionic moieties that are characteristic of binders to hydrophobic patches. Indeed, some GSMs have been reported as weak Aβ aggregation inhibitors and, vice-versa, potent aggregate binders such as X-34 and chrysamine G as moderate GSMs [19]. Noteworthy, the benzophenone cross-linking moiety used in the photo-affinity labeling studies with NSAID-type GSMs also shows strong structural similarity with fenofibrate and, in addition, with potent amyloid binders in the anthraquinone family [34], [35]. Hence, the cross-activities in γ-secretase modulation and/or APP-binding might reflect the high hydrophobicity of the putative targets and are not mutually exclusive in view of the available data [36], [37]. Clearly, we cannot exclude the possibility that NSAID-type GSMs target a different binding site than piperidine-based GSMs. Ebke et al have performed competition studies with the NSAID GSM sulindac sulfide and observed strongly reduced binding of their non-acidic GSM photo-probe indicative of overlapping binding sites [21]. Competition experiments are generally not able to distinguish between direct and allosteric competition for a specific binding site. Therefore, it remains possible that sulindac sulfide has a specific binding site within the substrate APP and reduces binding of the photo-probe to PSEN through allosteric interaction. However, because of the superior properties of the photo-probes based on second-generation GSMs, the overall structural similarities between NSAID GSMs and second generation acidic GSMs, the prior evidence from cell biological studies indicating a binding site of NSAID GSMs within PSEN, and the new evidence from competition experiments, the modulatory activity of GSMs appears to be strongly linked to interaction with PSEN. Future studies aided by tandem mass spectrometry might allow to fully define the binding sequence of GSM photo-probes [38]. In addition, fluorophore-coupled photo-probes that can be cross-linked in live cells might be useful to investigate whether GSMs target specific subcellular pools of γ-secretase. Such studies could achieve additional insights into the molecular mechanism of GSMs and Aβ42 generation, and might provide a basis for rational chemical design of GSMs.

## Materials and Methods

### Chemistry and Compounds

Compounds 1, 2, E-2012 and BB25 have been described previously [22]. Compound JNJ-40418677 was synthesized according to procedures described in WO2006112552 [39]. The chemical procedures for the GSM photo-probes used in this study are described here in exemplary fashion for compound AR243 *(2-(1-(7-((5-((3aS,4S,6aR)-2-oxohexahydro-1H-thieno[3,4-d]imidazol-4-yl)pentanoyl)oxy)-1-(4-(3(trifluoromethyl)-3H-diazirin-3-yl)phenyl)hept-2-yn-1-yl)-2-(4(trifluoromethyl)phenyl)piperidin-4-yl)acetic acid)*. The experimental procedures and characterization data for compounds BB25, AR80 and AR366 will be reported elsewhere.

All reactions were performed under an inert atmosphere of Argon, unless otherwise stated. ^1^H-NMR and ^13^C-NMR data were obtained on a Bruker Avance DPX 400 or DPX 500. Spectra were recorded at 298 K and the chemical shifts are expressed in ppm relative to solvent signals (^1^H-NMR: MeOD 3.31; ^13^C-NMR: MeOD 49.00). Preparative HPLC was performed on a Shimadzu LC-8A system using a gradient-system (H_2_O, 0,1% TFA and acetonitrile) at 5 ml/min on a 10x250 mm Supelco Ascentis C18 column. HRMS-spectra were recorded on a MAT 95 XL or MAT 90 mass spectrometer from Thermo Finnigan. All reagents were obtained from Sigma-Aldrich (Munich, Germany), Alfa-Aesar (Ward Hill, USA), Acros Organics (Geel, Belgium) or Fluorochem (Hadfield, UK) and used as received. In a glovebox under inert atmosphere were successively introduced in a pressure-tight vial: 120 mg of 4-(3-(trifluoromethyl)-3H-diazirin-3-yl)benzaldehyde (0,56 mmol)**,** 2-(trimethylsilyl)ethyl 2-(2-(4-(trifluoromethyl)phenyl)piperidin-4-yl)acetate (326 mg, 0,84 mmol) and Hex-5-yn-1-yl 5-((3aS,4S,6aR)-2-oxohexahydro-1H-thieno[3,4-d]imidazol-4-yl)pentanoate (273 mg, 0,84 mmol), together with activated mol-sieves (∼1 g / 10 ml solvent) and Copper(I)bromide (241 mg, 1,68 mmol). Toluene (6,3 ml) was added and the reaction was heated to 60°C for 2 days. The dark green suspension was filtered twice through celite, evaporated and filtered through a pad of silica-gel using DCM/MeOH 95/5. After evaporation, the obtained crude oil was dissolved in THF (15 ml) and TBAF (1 M in THF) (1,12 ml, 1,12 mmol) was slowly added at r.t. The reaction was allowed to proceed for 2 h before being diluted with EtOAc and water. The pH of the water layer was set neutral and washed with EtOAc. The organic layers were combined, washed with brine, dried over MgSO_4_, filtered and evaporated. The obtained crude oil was purified on prep-HPLC to afford AR243 as a white foam (42 mg, 9,3%).

**^1^H-NMR** (400 MHz, MeOD) δ = 1.39–1.91 (m, 12H), 1.99 (m, 1H), 2.12 (m, 1H), 2.21 (m, 1H), 2.31 (d, J = 6.8 Hz, 2H), 2.36 (t, J = 7.4 Hz, 2H), 2.55 (td, J_1_  = 6.8 Hz, J_2_ =  2.1 Hz, 2H), 2.68 (d, J = 12.9 Hz, 1H), 2.89 (dd, J_1_ = 12.9 Hz, J_2_ = 4.9 Hz, 1H), 2.98 (m, 1H), 3.10–3.20 (m, 2H), 4.19 (t, J = 6.5 Hz), 4.28 (dd, J_1_ = 8.1, J_2_ = 4.5 Hz, 1H), 4.38–4.50 (m, 2H), 4.90 (s, 1H), 7.32 (d, J = 8.1 Hz, 2H), 7.61 (d, J = 8.1 Hz, 2H), 7.76–7.89 (m, 4H).

**^13^C-NMR** (100 MHz, MeOD) δ = 19.14, 25.98, 25.99, 29.17, 29.47, 48.31, 29.71, 30.58, 33.31, 34.84, 40.30, 40.71, 41.02, 56.97, 59.34, 61.62, 63.39, 65.03, 68.43, 71.58. 94.82, 123.45 (q, J = 275.2 Hz), 125.39 (q, J = 275.5 Hz), 127.64 (q, J = 3.3 Hz), 127.95, 129.92 (broad), 131.43, 131.59, 132.49 (q, J = 32.5 Hz), 136.60, 143.46, 166.09, 175.40, 175.52 (diazirine-carbon not resolved).

**HRMS** (ESI-TOF) for C_39_H_43_F_6_N_5_O_5_SH^+^ (M+H) 808.2962, found 808.2938.

### Antibodies and cell lines

Monoclonal antibody IC16 raised against residues 1–15 of the human Aβ sequence, HRP-coupled carboxyl terminus-specific Aβ antibodies BAP24, BAP15 and BAP29 specific for Aβ40, Aβ42, and Aβ38, and polyclonal antibody CT15 against the last 15 C-terminal amino acids of human APP have been described previously [22], [40]. Monoclonal antibody PSN2 against a synthetic peptide corresponding to amino acids 31–56 of human PSEN1 was kindly provided by Dr. Hiroshi Mori [41]. The following commercial antibodies were used: polyclonal antibody against the C-terminal fragment of PSEN1 (Cat. No. 3622, Cell Signaling, Danvers, USA), polyclonal antibody against PEN-2 (Cat. No. 5451, Cell Signaling) and the C-terminal region of APH-1aL (Cat. No. PRB-550P, Covance, Princeton, USA), polyclonal antibody against the C-terminus of nicastrin (Cat. No. N1660, Sigma-Aldrich). HRP-coupled secondary antibodies against mouse or rabbit IgGs were purchased from Jackson ImmunoResearch Europe Ltd. (Suffolk, UK). CHO cells with stable co-expression of wild type human APP751 and human PSEN1 (PS70 cells), and N2a cells stably transfected with human APP harboring the “Swedish” mutation and human PSEN1, Nicastrin, APH-1a and PEN-2 (N2a-ANPP cells) have been described [26], [31]. HEK293T cells were purchased from GenHunter (Nashville, USA). All cell lines were maintained in Dulbecco's modified Eagle's medium with 10% fetal bovine serum, 1mM sodium pyruvate and 100 units/ml penicillin/streptomycin (Invitrogen).

### Dose-response experiments, IC_50_ determinations, and cytotoxicity assays

To determine IC_50_ values for the Aβ42-lowering activity of GSMs used in this study, PS70 cells were cultured in serum-containing medium and treated for 24 h with 8 increasing concentrations of each GSM or DMSO vehicle in triplicates. Aβ40, Aβ42 and Aβ38 peptide levels in conditioned cell culture supernatants were measured in a previously described sandwich ELISA assay [40]. For calculation of IC_50_ values, a non-linear curve-fit with variable slope model was applied to the results. Statistics were performed using GraphPad Prism 5.0 (GraphPad Software, San Diego, USA).

Cell viability was assessed using the alamarBlue^®^ reagent (Invitrogen). N2a-ANPP cells were seeded at low density in 96-well plates (4000 cells/well) and cultured for 24 h. The cells were then treated in duplicates with increasing concentrations of the respective compounds or DMSO as vehicle for an additional 24 h. 20 µl alamarBlue^®^ was added to cells cultured in 200 µl medium and incubated overnight. Absorbance was measured with a Paradigm™ microplate reader at 570 nm, using 600 nm as the reference wavelength, and percent viability of vehicle control was calculated.

### Photo-crosslinking and competition experiments

For photo-affinity labeling in living cells, N2a-ANPP were treated overnight with 0.2 µM of the GSM photo-probe AR243. Subsequently, cells were UV-irradiated on ice for 30 min at 365 nm and for 30 min at 302 nm. As negative controls, UV irradiation was omitted or cells were incubated with DMSO vehicle or compound AR80, which was structurally identical to AR243 but lacked the biotin moiety. Cells were washed and harvested in PBS and incubated with hypotonic buffer (10 mM Tris pH 7.4, 1x protease inhibitor mix) for 10 min on ice. To prepare cellular membranes, cells were passed 10 times through a 30G needle and centrifuged at 800×g, 10 min, 4°C. The post-nuclear supernatant was centrifuged at 18.000×g, 45 min, 4°C. The resulting membrane pellet was washed once with MES buffer (50 mM MES, 150 mM NaCl, 5 mM MgCl_2_, 5 mM CaCl_2_, pH 6.0), centrifuged at 18.000×g, 45 min, 4°C, and again dissolved in MES buffer. The total protein concentration of the membrane preparations was determined by BCA assay, concentrations and volumes of each sample were adjusted, and 5x RIPA buffer was added to a final concentration of 1x RIPA buffer (150 mM NaCl, 1.0% NP-40, 0.5% sodium deoxycholate, 0.1% SDS, 250 mM Tris HCl, pH 8.0). Solubilization was allowed to continue for 45 min at 4°C with gentle shaking. Insoluble debris was pelleted by centrifugation at 18.000×g, 45 min, 4°C. Protein concentrations were again determined and adjusted to equal levels prior to the addition of streptavidin magnetic beads (Invitrogen). Samples were incubated overnight at 4°C with gentle shaking, and the streptavidin beads were washed 3 times for 30 min with 1x RIPA buffer. Bound material was eluted with Laemmli buffer and incubation at 65°C for 10 min with intermittent vortexing. The eluted material was separated on 12% Bis-Tris gels and analyzed by Western blotting. Chemiluminescence was recorded with a LAS 3000 ECL camera system (Fuji Photo Film GmbH, Duesseldorf, Germany) or on CL-XPosure film (Fisher Scientific GmbH, Schwerte, Germany).

For photo-affinity labeling *in vitro*, membranes were prepared from N2a-ANPP or HEK293T cells as described above. Prior to the addition of compounds, membrane preparations were incubated with streptavidin magnetic beads to deplete endogenously biotinylated proteins. Membranes were then incubated with 0.5 µM of the GSM photo-probe AR243 or DMSO vehicle for 1 h at 4°C with gentle shaking. In competition experiments, the photo-probe was co-incubated with 100 µM of acidic or non-acidic GSM competitor compounds. 1% CHAPSO was used for solubilization. Samples were diluted to contain 0.25% CHAPSO and UV-irradiated. After the photo-crosslinking, to ensure exposure of the bound biotin-tagged GSM, 1x RIPA buffer was added to unfold/denature proteins. The pull-down and analysis of labeled target proteins using streptavidin magnetic beads was performed as described above.

## Supporting Information

Figure S1 **The photo-probe AR366 inversely modulates γ-secretase and targets the N-terminal fragment of PSEN1.** (**A**) Chemical structure of AR366, an inverse GSM photo-probe. AR366 was obtained through esterification of the carboxylic acid group in AR243. Note that, in contrast to AR243, the biotin moiety for affinity purification was placed on the alkynyl side chain via an amide bond. AR366 increased Aβ42 levels with an IC_50_ = 1.1 µM. (**B**) Incubation of solubilized membranes from N2a-ANPP cells with 500 nM of AR366 resulted in photo-affinity labeling of PSEN1-NTF suggesting that acidic GSMs and inverse GSMs either target the same or a closely related binding site. Input represents 0.02% of the total membrane material.
(TIF)
