# Supplementary figures and images for: Presenilin Is the Molecular Target of Acidic γ-Secretase Modulators in Living Cells
Source: PLoS One. 2012 Jan 6;7(1):e30484. doi: 10.1371/journal.pone.0030484 (PMC3253113; doi:10.1371/journal.pone.0030484)

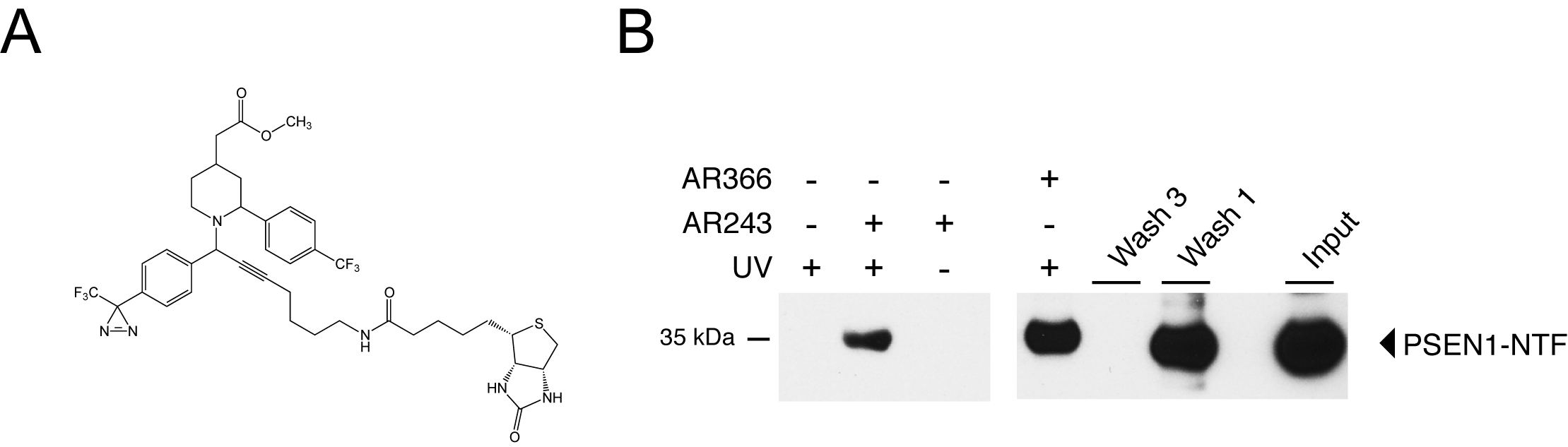

Supplement: Figure S1 — The photo-probe AR366 inversely modulates γ-secretase and targets the N-terminal fragment of PSEN1. (A) Chemical structure of AR366, an inverse GSM photo-probe. AR366 was obtained through esterification of the carboxylic acid group in AR243. Note that, in contrast to AR243, the biotin moiety for affinity purification was placed on the alkynyl side chain via an amide bond. AR366 increased Aβ42 levels with an IC50 = 1.1 µM. (B) Incubation of solubilized membranes from N2a-ANPP cells with 500 nM of AR366 resulted in photo-affinity labeling of PSEN1-NTF suggesting that acidic GSMs and inverse GSMs either target the same or a closely related binding site. Input represents 0.02% of the total membrane material. (TIF) [file pone.0030484.s001.tif]
